# Toxicity of Insecticides on Various Life Stages of Two Tortricid Pests of Cranberries and on a Non-Target Predator

**DOI:** 10.3390/insects7020015

**Published:** 2016-04-15

**Authors:** Cesar Rodriguez-Saona, Andrea Carolina Wanumen, Jordano Salamanca, Robert Holdcraft, Vera Kyryczenko-Roth

**Affiliations:** 1 P.E. Marucci Center for Blueberry & Cranberry Research & Extension, Rutgers University, 125A Lake Oswego Rd., Chatsworth, NJ 08019, USA; rholdcra@rci.Rutgers.edu (R.H.); vera_k_roth@yahoo.com (V.K.-R.); 2 Departamento de Protección de Cultivos, Escuela Técnica Superior de Ingeniería Agrónoma, Universidad Politécnica de Madrid, Avenida Complutense s/n, Madrid 28040, Spain; ancawa@gmail.com; 3 Departmento de Entomologia, Universidade Federal de Lavras, PO Box 3037, CEP 37200-000 Lavras, Minas Gerais, Brazil; jordanosalamanca@gmail.com

**Keywords:** IPM, reduced-risk insecticides, *Sparganothis sulfureana*, *Choristoneura parallela*, conservation biological control, *Orius insidiosus*

## Abstract

Laboratory and extended laboratory bioassays were conducted to determine the residual toxicities of various insecticides against two key pests of cranberries, *Sparganothis sulfureana* and *Choristoneura parallela* (Lepidoptera: Tortricidae), and their non-target effects on the predatory *Orius insidiosus* (Hemiptera: Anthocoridae). The effects of nine insecticides with different modes of action on *S. sulfureana* and *Ch. parallela* eggs, larvae, and adults were tested in the laboratory, while the efficacy of a post-bloom application on larval mortality and mass of these pests and on adult *O. insidiosus* was evaluated in extended laboratory experiments. The organophosphate chlorpyrifos and the spinosyn spinetoram provided long-lasting (seven-day) control against all stages of both pests. The growth regulator methoxyfenozide and the diamides chlorantraniliprole and cyantraniliprole had strong (1–7 days) larvicidal, particularly on young larvae, and growth inhibitory activity, but only the diamides were adulticidal. Among neonicotinoids, acetamiprid had stronger ovicidal and adulticidal activity than thiamethoxam, showing within-insecticide class differences in toxicities; however, both were weak on larvae. Lethality of novaluron and indoxacarb was inconsistent, varying depending on species and stage. Chlorpyrifos was most toxic to *O. insidiosus*. These results show species- and stage-specific toxicities, and greater compatibility with biological control, of the newer reduced-risk classes of insecticides than older chemistries.

## 1. Introduction

The use of insecticides in agroecosystems is constantly changing in response to the needs for products with greater public safety and reduced environmental risks [1]. This has led to the development of new classes of chemicals with novel modes of action [2,3]. Moreover, the implementation of the Food Quality Protection Act (FQPA) by the Environmental Protection Agency (EPA) in the United States of America (USA) [4] and the European Directive 2009/128/EC on sustainable use of pesticides and regulation in Europe (REGULATION (EC) No. 1107/2009) [5] has resulted in increased restrictions on the use of broad-spectrum insecticides, and their replacement with reduced-risk insecticides [1,6]. Reduced-risk insecticides have lower mammalian toxicity, are safer to the environment, and are more compatible with integrated pest management (IPM) than broad-spectrum insecticides [6]. However, insecticides, even those belonging to the same class, may have different toxicological effects on pests depending on their mode of action, residual activity, and insect species, developmental stage, and gender (e.g., [7,8]).

The American cranberry, *Vaccinium macrocarpon* Aiton, is a fruit native to wetland habitats of the northeastern USA that, under commercial production, grows on so-called bogs, marshes, or beds [9]. In 2014, the total cranberry production in the USA was 994,082 tons with a total value of $254.4 million; New Jersey (USA) was ranked third in the nation with a total production of 77,160 tons valued at $22.7 million [10]. Cranberries produce various phenolic compounds in leaves and fruit [11], which makes them more resistant to insect feeding [12,13]. Despite such resistance, pest pressure may increase when cranberries are grown commercially as large-scale monocultures [14]. New Jersey cranberries are grown in the Pine Barrens, an ecologically-sensitive area; thus, pest management with insecticides must minimize environmental and non-target risks [15].

Sparganothis fruitworm, *Sparganothis sulfureana* (Clemens) (Lepidoptera: Tortricidae), and the spotted fireworm, *Choristoneura parallela* (Robinson) (Lepidoptera: Tortricidae), are two key pests of cranberries across most major cranberry-producing regions and in New Jersey, respectively. Both species are native to the USA and have similar life histories [16,17,18,19]. Both complete two generations per year. In the spring, overwintered larvae feed on new plant growth [17]. First generation adults emerge in early summer (June) and deposit egg clusters on cranberry leaves or on weeds inside bogs in the case of *S. sulfureana* and *Ch. parallela*, respectively. The second generation larvae develop in early summer (June–July); they feed on leaves, but may also feed on developing berries when available [17]. Due to direct injury to young fruit, feeding by larvae from this generation are the most detrimental to the crop. Additionally, older instars are protected from insecticides while feeding inside the fruit, so timing of chemical applications is critical to achieve good control. Second-generation adults emerge in late summer (August). Early-instar larvae from the late-summer generation overwinter in cranberry bogs.

Although implementation of IPM in cranberries has reduced overall pesticide use [20], control methods for *S. sulfureana* and *Ch. parallela* still rely quite heavily on insecticides. There have been studies on alternative control methods for managing both pests, such as behavioral [21] and biological control [18,22]; however, so far, these have not been proven economically feasible or as effective as chemical control. Both generations of *S. sulfureana* and *Ch. parallela* are controlled most effectively with insecticides targeting early-instar larvae, principally in spring when they are emerging from diapause or in early summer before they enter the fruit [17,23]. In the 1950s, Marucci [24] pointed out that broad-spectrum insecticide applications in cranberries negatively affect the natural enemies of these pests. Therefore, in order to develop sound IPM programs that are compatible with biological control in cranberries, the toxicities of old (broad-spectrum) and newer (reduced-risk) insecticide chemicals need to be evaluated against economically-important pests such as *S. sulfureana* and *Ch. parallela*, as well as their non-target effects on the pests’ natural enemies.

In the present study, a series of laboratory and extended laboratory experiments was conducted to examine the fresh (*i.e.*, soon after application) and aged-residual toxicological effects of various insecticides, with different modes of action, on eggs, 1st and 3rd instar larvae, and adults of *S. sulfureana* and *Ch. parallela*, and their non-target effects on the predatory insidiosus flower bug *Orius insidiosus* (Say) (Hemiptera: Anthocoridae). We hypothesized that: (a) the toxicities of insecticides (broad-spectrum and reduced-risk) against these two cranberry pests vary by species, developmental stage, and gender; and (b) reduced-risk insecticides have lower toxicities on non-targets than broad-spectrum insecticides. Specifically, we asked: (1) Do different classes of insecticides differentially affect egg, larval, and adult survival and larval growth of *S. sulfureana* and *Ch. parallela*? (2) What are the residual activities of these insecticides? (3) Are there differences between adult males and females in their susceptibility to insecticides? Finally, (4) How do these insecticides affect *O. insidiosus* survival? In the summer months (late June–early August), all three stages (eggs, larvae, and adults) of these pests can overlap, whereas in the spring (May) only the larval stages are present [17]; therefore, these stages are the primary targets of insecticide applications. *Orius insidiosus* is also abundant in July in cranberries [25]. Thus, these studies have important implications for managing *S. sulfureana* and *Ch. parallela* and the conservation of biological control agents during the cranberry growing season when insecticide applications are most critical.

## 2. Experimental Section

### 2.1. Insects

Eggs, larvae, and adults of *S. sulfureana* and *Ch. parallela* were obtained from laboratory colonies at the Rutgers P.E. Marucci Center (Chatsworth, NJ, USA). The colonies have been kept for over 10 years; wild larvae have been added to the colonies every year to maintain genetic integrity. Adults were kept inside air-filled plastic bags, that were also used as an oviposition substrate, and provided with cotton balls saturated with 10% *m*/*v* sugar-water solution. Egg masses laid on the bag’s interior surface were collected and placed in 5 cm Petri dishes (Thermo Fisher Scientific Inc., Pittsburgh, PA, USA), on a slightly moistened (with distillate water) piece of filter paper (Whatman No. 4; Maidstone, Kent, UK). The egg masses were kept in an incubator at 25 ± 5 °C, 50%–60% RH, and 10:14 (L:D), and monitored daily until they hatched. Subsequently, neonate larvae were placed in 28.3 g plastic shot glasses (four larvae per glass) (Frontier Agricultural Sciences, Newark, DE, USA), containing commercial “stonefly *Heliothis*” diet (Ward’s Natural Science, Rochester, NY, USA). Once larval development was completed, pupae were removed from the diet and placed in 453.6 g plastic deli containers (Placon, Madison, WI, USA), lined with Kimwipe paper (11.4 cm × 21.3 cm; Fischer Scientific), until the adults emerged. The same rearing protocol was used for both species.

*Orius insidiosus* adults were obtained from Rincon-Vitova Insectaries, Inc. (Ventura, CA, USA). *Orius* spp. are generalist predators of many lepidopteran pests (e.g., [26,27]); thus, *S. sulfureana* and *Ch. parallela* eggs and young instar larvae can serve as suitable prey items to *O. insidiosus* in cranberries.

### 2.2. Treatments

Insecticide treatments were prepared at a concentration equivalent to the labeled field application rate (Table 1). Insecticides were selected to represent a wide array of modes of action and chemical classes. These insecticides can be broadly classified into: broad-spectrum organophosphates (chlorpyrifos), organophosphate-replacements (novaluron, acetamiprid, and thiamethoxam), and reduced-risk (chlorantraniliprole, spinetoram, cyantraniliprole, methoxyfenozide, and indoxacarb) [28]. All insecticides evaluated, except for cyantraniliprole (which is under the process of registration), are currently registered for use in cranberries in the USA [29]. Chlorpyrifos was used as the grower standard. All experiments were conducted at the Rutgers P.E. Marucci Center for Blueberry and Cranberry Research and Extension in Chatsworth, New Jersey, NJ, USA.

### 2.3. Laboratory Assays

A series of experiments was conducted in 2011 and 2013 to evaluate the effect of various insecticides (Table 1) on *S. sulfureana* and *Ch. parallela* eggs, larvae (neonates and 3rd instars), and adults (males and females). Treatments were applied using a Potter precision spray tower (Burkard Scientific, Uxbridge, UK), with an output equivalent to 280 L/hectare (30 gallons/acre), diluted in deionized water (spray volume = 28 mL/m^2^). In addition to the insecticides in Table 1, experiments included an untreated control (deionized water alone) for a total of 10 treatments. The spray tower was calibrated between treatments.

#### 2.3.1. Egg Toxicity

The ovicidal activity of insecticides (Table 1) was measured by placing 7 egg masses of either *S. sulfureana* or *Ch. parallela* in a Petri dish (5 cm diameter; Fisher Scientific) lined with a slightly moistened filter paper (Whatman No. 4). The number of eggs per mass was counted before treatment. Treatments were sprayed directly on top of the egg masses and allowed to dry for 30 min. Egg masses were placed in individual Petri dishes that were sealed with Parafilm M (Pechiney Plastic Packaging, Chicago, IL, USA), and then placed in an incubator at 25 ± 5 °C; 50%–60% RH and 10:14 (L:D). Eggs were checked daily 10–14 days after treatment to monitor for their viability (number of developed eggs) and egg hatch (number of emerged larvae); the eggs of both pest species take about 10–12 days to hatch [17,30,31]. Viability of eggs was determined under a microscope by recording the number of eggs that changed coloration (for *S. sulfureana* from light green-yellow to dark green-black color; and for *Ch. parallela* from yellow-orange to black), which is indicative of egg development; non-viable eggs were those that did not change in color. Each Petri dish was considered as a replicate, and each treatment was replicated 10 times; *S. sulfureana* and *Ch. parallela* were tested separately.

#### 2.3.2. Larval Toxicity

The toxicity of insecticides to *S. sulfureana* and *Ch. parallela* larvae was evaluated in Petri dishes (4 cm diameter; Fisher Scientific) half-filled with artificial diet (see above, Section 2.1). Insecticides were applied directly to the surface of the diet, and residues were allowed to dry for 2 h before placing the larvae in the Petri dishes. Either two 1st instars or one 3rd instar larvae were then placed on the surface of the dried diet in each Petri dish using a fine paint brush. Larvae were exposed to treated diet at 0 (*i.e.*, 2 h after treatment) and 3 days after treatment (3 DAT); with a new set of insects and diet for each date. To prevent larvae from escaping, treated Petri dishes were sealed with Parafilm M (Pechiney Plastic Packaging), and then placed in an incubator at the conditions described above. Larval mortality was recorded 7 days after exposure to treated diet. Percent mortality was calculated by adding the number of dead and missing larvae (assumed to have died and broken down). Each Petri dish was considered as a replicate, and each treatment and residue age was replicated 10 times; each moth species was tested separately.

#### 2.3.3. Adult Toxicity

To evaluate the toxicity of insecticides on *S. sulfureana* and *Ch. parallela* adults, the inner surfaces of Petri dishes (5 cm diameter; Fisher Scientific) were treated with insecticides and allowed to dry for 2 h. After drying, one adult was placed inside each Petri dish together with a small piece of cotton ball saturated with a 10% *m*/*v* sugar-water solution to serve as food. Petri dishes were sealed with Parafilm M (Pechiney Plastic Packaging), and then placed in an incubator (as described above, Section 2.1.). Adults were exposed to treated surfaces at 0, 3, and 7 DAT; with a new set of insects and dishes for each date. Adult mortality was recorded 48 h after insecticide exposure. Insects that were moving but failed to remain upright were considered moribund (*i.e.*, knocked down), whereas insects that exhibited no or very little movement when touched with a probe were considered dead. Each treatment was replicated 10 times for each species and residue age (5 replicates for each gender).

### 2.4. Extended Laboratory Experiments

Because the substrate on which an insecticide is deposited can affect its activity (e.g., [32]), as can the conditions during residue aging, extended laboratory trials were conducted to evaluate the mortality and growth effects of various insecticides (Table 1) on *S. sulfureana* and *Ch. parallela* 1st and 3rd instar larvae exposed to residues on cranberry foliage from 2012 to 2015. In addition to the insecticides listed in Table 1, an untreated control was included for a total of 10 treatments. Although most treatments were tested in 2012 (Table 1), because of limited availability of larvae for bioassays, experiments were expanded across 4 years. In all years, however, chlorpyrifos (used as grower standard) and untreated control treatments were included as reference for comparison. The experiments were done in a mature cranberry bog cv. “Early Black” located at the Rutgers P.E. Marucci Center. The bog was managed under standard commercial conditions (e.g., fertilizer, fungicides, *etc.*); however, no insecticides were applied. Treatments were applied to 60 cm × 60 cm plots, separated by a 15 cm buffer zone, and were replicated 4 times. Treatment applications were made from mid-July through early-August (post-bloom) with a CO_2_ backpack sprayer (R&D Sprayers, Opelousas, LA, USA), using a 1 L plastic bottle, and done early in the morning at wind speeds of <4 km/h to avoid drift; during the spray the sprayer’s nozzle was kept within 30 cm from the top of the canopy to further avoid drift. No precipitation was recorded during the experiments. The sprayer was calibrated to deliver 467 L per hectare (50 gallons/acre) at 30 psi, using a single TeeJet VS nozzle (size 110015; Spraying Systems Co, Wheaton, IL, USA), yielding 47 mL per cm^2^. This application volume is within the range recommended for optimal canopy deposition with insecticides under commercial conditions in cranberries. After treatment, foliage (uprights) from field plots was collected, placed in plastic bags, and brought to the laboratory 1, 3, and 7 DAT. Foliage from all four plots of each treatment was combined before selecting uprights used in bioassays. In the laboratory, 3 or 4 uprights (each ~15 cm in length) from a given treatment were inserted into water-filled florists’ water picks that were secured on Styrofoam trays. Uprights were randomly selected from all foliage collected for that treatment. The top of uprights (containing foliage and fruits) was enclosed in a ventilated 40 dram plastic snap cap vial (3 cm × 5 cm) to prevent larvae from escaping, while the lower parts (bare stems only) passed through a hole in the vial bottoms and were placed inside the water picks. Three 1st instars or one 3rd instar larvae were placed inside each vial. Vials were placed on a light bench in the laboratory (25 ± 5 °C, 50%–60% RH and 15:9 L:D). Larval mortality (% of dead and missing larvae) and mass of surviving 3rd instar larvae were assessed after 7 days. Larval mass was recorded for 3 and 7 DAT using a Mettler-Toledo AE50 analytical balance of 0.1 mg resolution (Mettler Instrument Corp., Hightstown, NJ, USA). There were 10 vials per treatment for each species and residue age, and each vial was considered a replicate.

### 2.5. Non-Target Effects

The toxicity of various insecticides on *O. insidiosus* adults was tested in an extended laboratory experiment. All insecticides listed in Table 1 were tested in this experiment, except for thiamethoxam, and an untreated control for a total of 9 treatments. The experiment was conducted in July 2012 and followed the same protocol as described above (see Section 2.4). Residual activity was evaluated 3 and 7 DAT. Groups of 5 *O. insidiosus* adults (mix of males and females) were placed in each vial, mortality was recorded 24 h after exposure, and there were six vials per treatment and residue age (replicates).

### 2.6. Statistical Analysis

Mean percent mortality of eggs, larvae, and adults of *S. sulfureana* and *Ch. parallela* for each treatment at each time check (residual activity) was arcsine square-root transformed. Similarly, mean percent mortality of *O. insidiosus* adults for each treatment and time check was arcsine square-root transformed. Mass data were natural log (ln)-transformed. Transformed mortality and mass data were analyzed using analysis of variance (ANOVA), with treatment, instar, time check (date), or gender, and their interactions, as factors [33]. To simplify our analysis, extended laboratory data for each species and instar were combined across years because larval mortality in untreated control and chlorpyrifos (grower standard) treatments did not change among years (*p* > 0.05) [34]. To avoid pseudo-replication, if more than 1 larva were placed per vial (replicate), larval mortality and mass data for each vial were averaged to obtain a mean value for each vial prior to analysis. Untransformed percent data are shown in the Figures. Mean differences were compared using Tukey’s test (α = 0.05) after a significant *F*-test.

## 3. Results

### 3.1. Laboratory Assays

#### 3.1.1. Egg Toxicity

**Figure 1:**
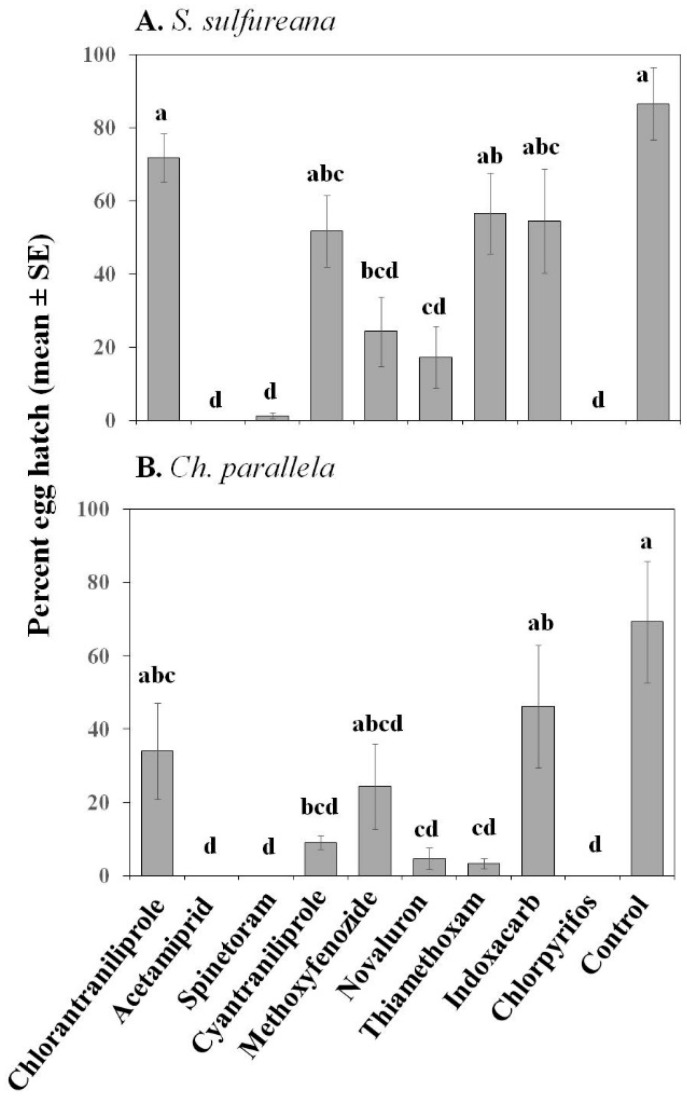
Effect of various insecticides on egg hatch for *Sparganothis sulfureana* (**A**) and *Choristoneura parallela* (**B**). Bars with the same letter are not significantly different at α = 0.05.

For both pest species, the insecticides evaluated here had no effect on percent egg viability (Table 2); however, some insecticides had a negative impact on egg hatch (Table 2; Figure 1). For both species, acetamiprid, spinetoram, novaluron, and chlorpyrifos had the highest ovicidal activity; while chlorantraniliprole and indoxacarb were the least toxic to *S. sulfureana* and *Ch. parallela* eggs (Figure 1). The insect growth regulator methoxyfenozide had intermediate ovicidal toxicity against both species. There were, however, some discrepancies between species: cyantraniliprole and thiamethoxam were relatively harmless to *S. sulfureana* eggs but toxic to *Ch. parallela* eggs (Figure 1).

#### 3.1.2. Larval Toxicity

Insecticide treatment, larval instar, and DAT affected *S. sulfureana* and *Ch. parallela* larval mortality (Table 2). However, these factors interacted in different ways to affect both species, indicating some species-specific effects. For instance, all factors interacted to affect *S. sulfureana* larval mortality, while only treatment and instar interacted to affect *Ch. parallela* larval mortality (Table 2).

**Figure 2:**
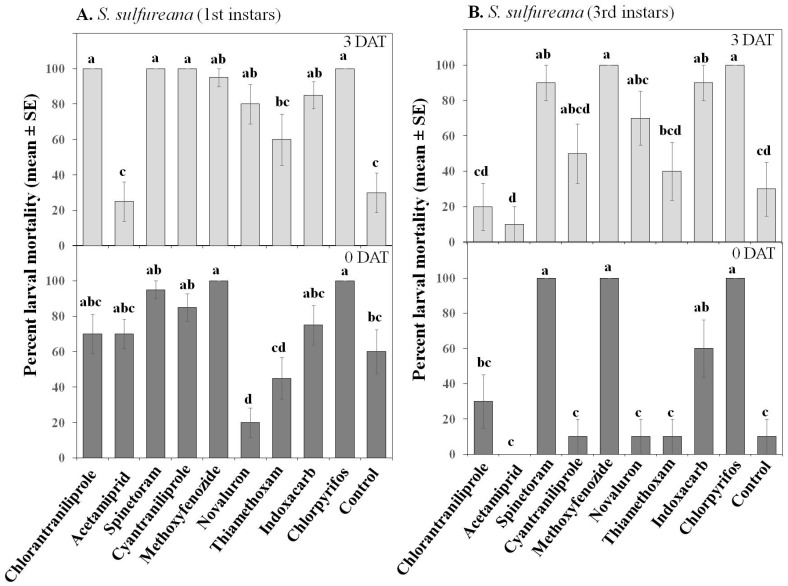
Effect of various insecticides on *Sparganothis sulfureana* 1st (**A**) and 3rd (**B**) instar larvae after exposure to 2 h (0 DAT) and 3 DAT laboratory-aged residues. DAT = days after treatment. Bars with the same letter are not significantly different at α = 0.05.

**Figure 3:**
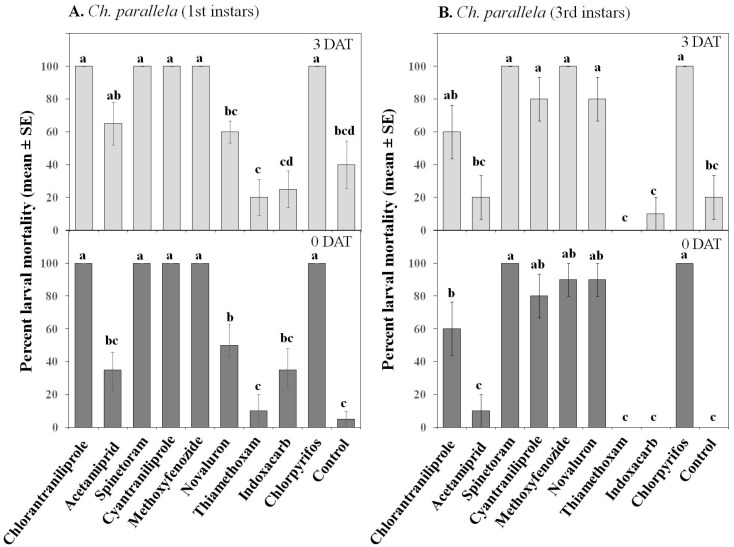
Effect of various insecticides on *Choristoneura parallela* 1st (**A**) and 3rd (**B**) instar larvae after exposure to 2 h (0 DAT) and 3 DAT laboratory-aged residues. DAT = days after treatment. Bars with the same letter are not significantly different at α = 0.05.

For both species, chlorantraniliprole, spinetoram, cyantraniliprole, methoxyfenozide, and chlorpyrifos were highly toxic to 1st instar larvae, and their toxicity lasted for at least 3 DAT (Figure 2 and Figure 3). In contrast, acetamiprid, novaluron, and thiamethoxam had weak toxicities against *S. sulfureana* and *Ch. parallela* 1st instar larvae and had inconsistent residual activities (Figure 2). Interestingly, indoxacarb had higher toxicity to *S. sulfureana* 1st instar larvae than to *Ch. parallela* 1st instar larvae.

As expected, 3rd instar larvae of both species were more resistant to insecticides than 1st instar larvae, but this effect depended on the insecticide (significant Treatment × Instar interaction for both species; Table 2). In fact, only spinetoram, methoxyfenozide, and chlorpyrifos were highly toxic to 3rd instar *S. sulfureana* and *Ch. parallela* larvae at 0 and 3 DAT (Figure 2 and Figure 3). The neonicotinoids acetamiprid and thiamethoxam were weak against 3rd instars. Contrasting both species, indoxacarb was only toxic to *S. sulfureana* 3rd instar larvae, while the diamides chlorantraniliprole and cyantraniliprole and the chitin inhibitor novaluron were more toxic to *Ch. parallela* 3rd instar larvae (Figure 2 and Figure 3).

#### 3.1.3. Adult Toxicity

**Figure 4:**
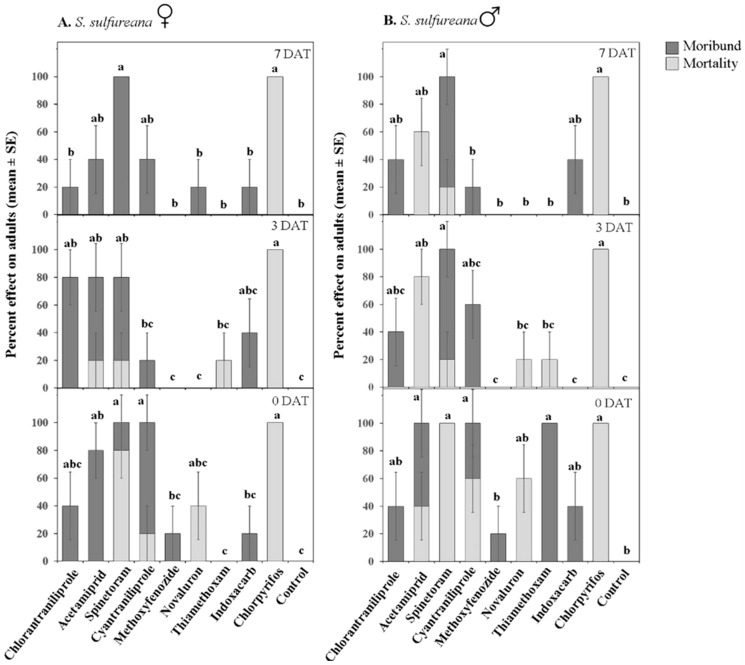
Effect of various insecticides on *Sparganothis sulfureana* adult females (**A**) and males (**B**) after exposure to 2 h (0 DAT), 3 DAT, and 7 DAT laboratory-aged residues. DAT = days after treatment. Dark portions of bars represent percent moribund (*i.e.*, insects that were moving but failed to remain upright); light portions represent percent mortality (*i.e.*, insects that exhibited no or very little movement when touched with a probe). Bars with the same letter are not significantly different at α = 0.05.

Insecticides affected, albeit differently, *S. sulfureana* and *Ch. parallela* adult mortality (significant Treatment effect; Table 2). For *S. sulfureana*, acetamiprid, spinetoram, cyantraniliprole, and chlorpyrifos were highly toxic (80%–100% moribund and/or dead) to both sexes at 0 DAT, while chlorantraniliprole, methoxyfenozide, novaluron, and indoxacarb had weak adulticidal activities (Figure 4). Only chlorpyrifos and spinetoram (0 DAT) caused high adult mortality; all other insecticides caused high adult knock-down. Thiamethoxam was higly toxic to *S. sulfureana* males but not females (significant Treatment × Sex interaction; Table 2). Acetamiprid, spinetoram, and chlorpyrifos remained toxic (≥80% moribund and/or dead) against adults 3 DAT; whereas only spinetoram and chlorpyrifos maintained high toxicity 7 DAT (significant Treatment × Date interaction; Table 2).

**Figure 5:**
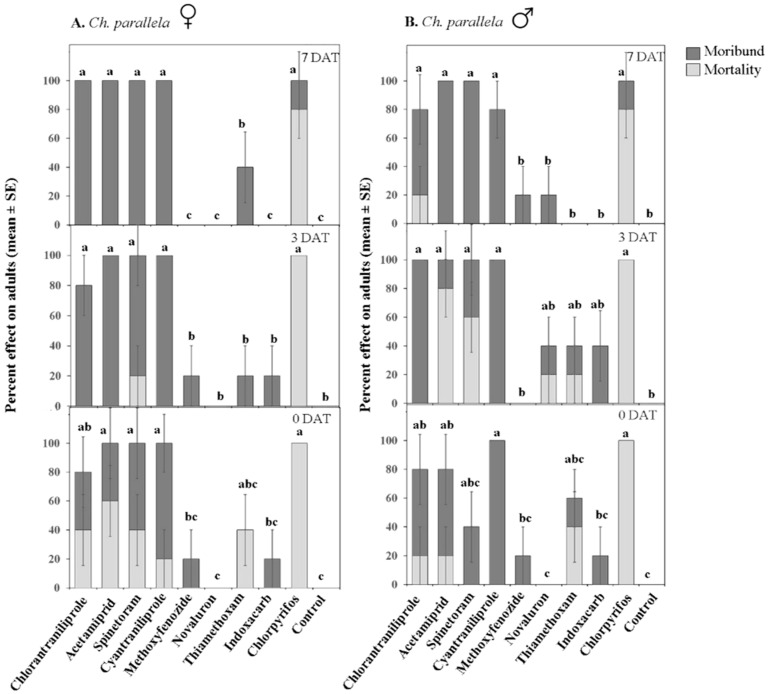
Effect of various insecticides on *Choristoneura parallela* adult females (**A**) and males (**B**) after exposure to 2 h (0 DAT), 3 DAT, and 7 DAT laboratory-aged residues. DAT = days after treatment. Dark portions of bars represent percent moribund (*i.e.*, insects that were moving but failed to remain upright); light portions represent percent mortality (*i.e.*, insects that exhibited no or very little movement when touched with a probe). Bars with the same letter are not significantly different at α = 0.05.

For *Ch. parallela*, chlorantraniliprole, acetamiprid, spinetoram, cyantraniliprole, and chlorpyrifos were toxic to both sexes, whereas methoxyfenozide, novaluron, thiamethoxam, and indoxacarb were all weak against *Ch. parallela* adults (Figure 5). Chlorantraniliprole, acetamiprid, spinetoram, cyantraniliprole, and chlorpyrifos remained toxic 7 DAT against both sexes; and, all of these insecticides, except for chlorpyrifos that caused >80% adult mortality, caused high adult know-down.

### 3.2. Extended Laboratory Experiments

Insecticide class, instar, and residual age, and their two-way interaction, had an effect on *S. sulfureana* and *Ch. parallela* larval mortality (Table 3). For *S. sulfureana*, the effect of insecticide on larval mortality was influenced by instar and residual age (significant three-way interaction; Table 3).

**Figure 6:**
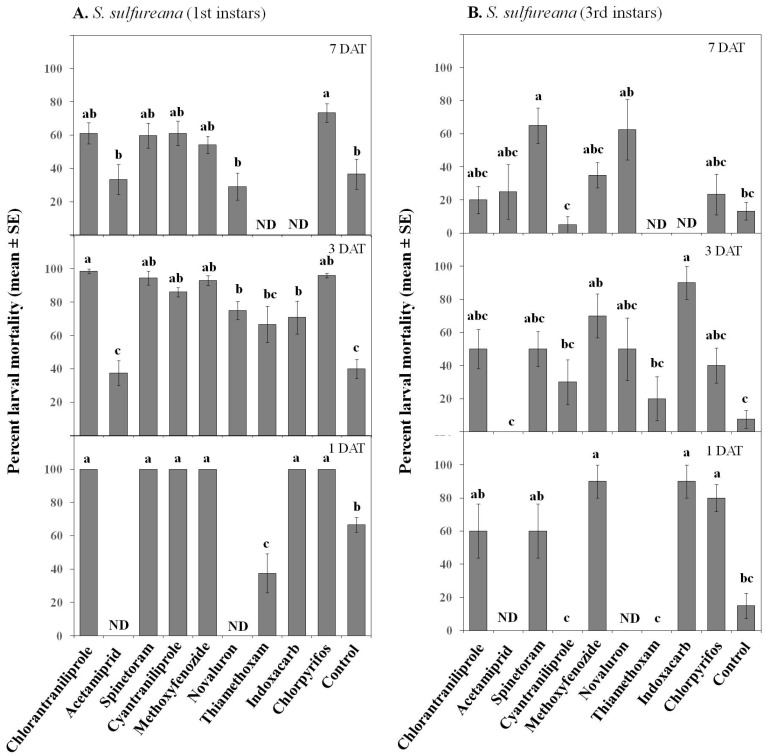
Effect of various insecticides on *Sparganothis sulfureana* 1st (**A**) and 3rd (**B**) instar larvae after exposure to 1, 3, and 7 DAT field-aged residues. DAT = days after treatment. ND = not determined. Bars with the same letter are not significantly different at α = 0.05.

For *S. sulfureana*, chlorantraniliprole, spinetoram, cyantraniliprole, methoxyfenozide, indoxacarb, and chlorpyrifos were highly toxic to 1st instar larvae; in contrast, novaluron had intermediate toxicity, while the neonicotinoids acetamiprid and thiamethoxam had weak toxicities against *S. sulfureana* 1st instar larvae (Figure 6A). Although the toxicities of insecticides declined with field aging, chlorantraniliprole, spinetoram, cyantraniliprole, methoxyfenozide, indoxacarb, and chlorpyrifos were still toxic 3 DAT as compared with controls. Only chlorpyrifos remained toxic 7 DAT. Fewer insecticides, *i.e.*, methoxyfenozide, indoxacarb, and chlorpyrifos, were highly toxic to 3rd instars 1 DAT (Figure 6B), while chlorantraniliprole, spinetoram, and novaluron had intermediate insecticidal toxicity and acetamiprid, cyantraniliprole, and thiamethoxam had weak to zero toxicity. As expected, all insecticides lost toxicity with residual age; however, indoxacarb, spinetoram, methoxyfenozide, and novaluron remained toxic 3–7 DAT (Figure 6B).

**Figure 7:**
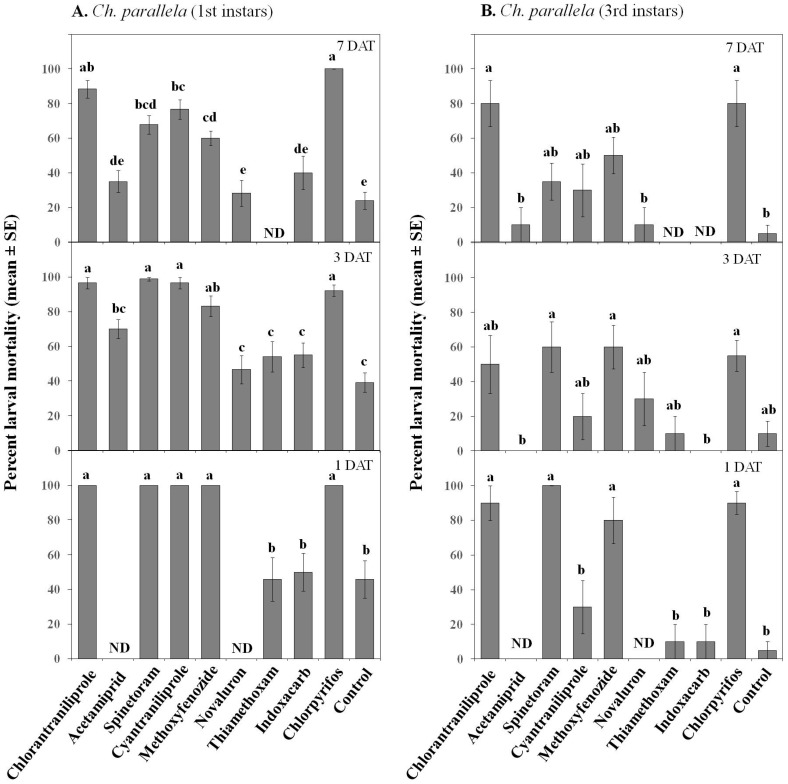
Effect of various insecticides on *Choristoneura parallela* 1st (**A**) and 3rd (**B**) instar larvae after exposure to 1, 3, and 7 DAT field-aged residues. DAT = days after treatment. ND = not determined. Bars with the same letter are not significantly different at α = 0.05.

For *Ch. parallela*, chlorantraniliprole, spinetoram, cyantraniliprole, methoxyfenozide, and chlorpyrifos were highly toxic to 1st instar larvae; in contrast, acetamiprid, novaluron, thiamethoxam, and indoxacarb had weaker toxicities (Figure 7A). Chlorantraniliprole, spinetoram, cyantraniliprole, methoxyfenozide, and chlorpyrifos remained toxic 3–7 DAT as compared with controls. Only chlorantraniliprole, spinetoram, methoxyfenozide, and chlorpyrifos were highly toxic to 3rd instars 1 DAT (Figure 7B), but all (except for chlorpyrifos) had reduced toxicities 3–7 DAT. In contrast, acetamiprid, cyantraniliprole, novaluron, thiamethoxam, and indoxacarb had weak to zero toxicity on 3rd instar *Ch. parallela* larvae.

**Figure 8:**
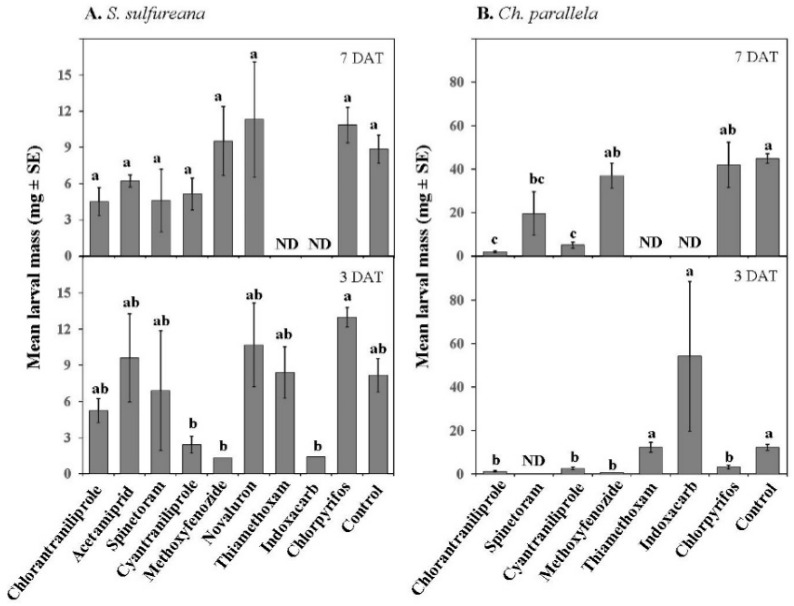
Effect of various insecticides on *Sparganothis sulfureana* (**A**) and *Choristoneura parallela* (**B**) 3rd instar larval mass after exposure to 3 and 7 DAT field-aged residues. DAT = days after treatment. ND = not determined. Bars with the same letter are not significantly different at α = 0.05.

In addition, insecticide treatment had an effect on the mass of surviving *S. sulfureana* and *Ch. paralella* larvae (Table 3). Although cyantraniliprole, methoxyfenozide, and indoxacarb reduced *S. sulfureana* larval mass 1 DAT, this effect was not significantly different from controls; no effect of insecticide on larval mass was found 7 DAT (Figure 8). For *Ch. parallela*, chlorantraniliprole, cyantraniliprole, methoxyfenozide, and chlorpyrifos reduced mass of surviving larvae 1 DAT, but only chlorantraniliprole, spinetoram, and cyantraniliprole reduced larval mass 7 DAT, as compared with controls (Figure 8).

### 3.3. Non-Target Effects

**Figure 9:**
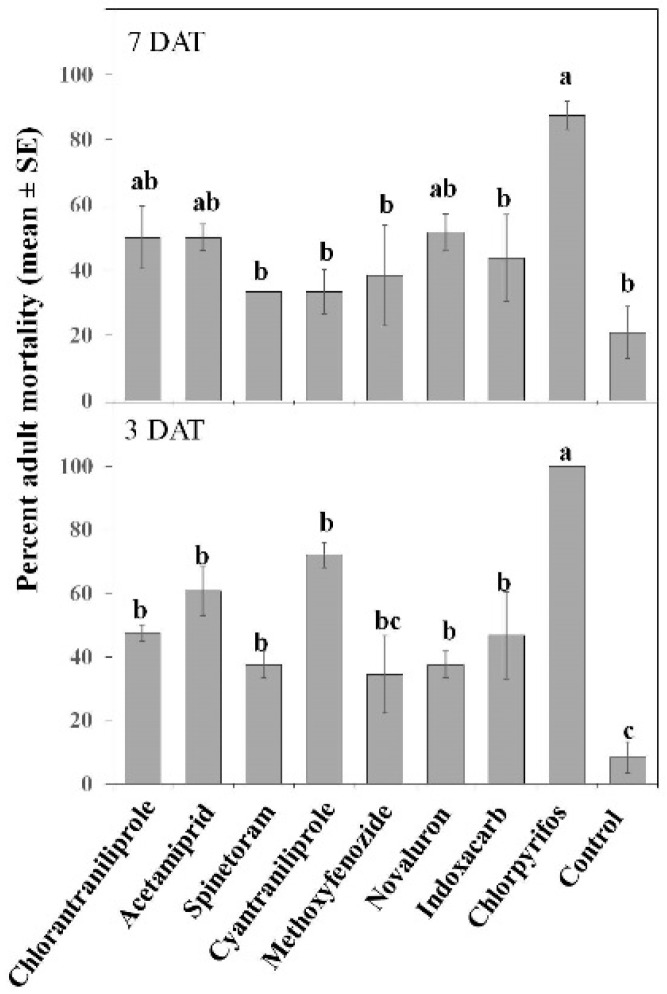
Effect of various insecticides on *Orius insidiosus* adults after exposure to 3 and 7 DAT field-aged residues. Bars with the same letter are not significantly different at α = 0.05.

Insecticides had different toxicities on *O. insidiosus* adults (significant Treatment effect; *F* = 17.85; *df* = 8, 54; *p* ≤ 0.001). Mortality of *O. insidiosus* was highest for chlorpyrifos (Figure 9). In contrast, chlorantraniliprole, acetamiprid, spinetoram, cyantraniliprole, novaluron, thiamethoxam, and indoxacarb had intermediate toxicities (Figure 8), with acetamiprid and cyantraniliprole being the most toxic and spinetoram and novaluron being the least toxic among them 3 DAT. The insect growth regulator methoxyfenozide had the lowest toxicity on *O. insidiosus* 3 DAT and its effect was not significantly different from the control (Figure 8). Although there was no effect of DAT (no significant Date effect; *F* = 1.40; *df* = 1, 54; *p* = 0.24), there was a significant Treatment × Date interaction (*F* = 2.13; *df* = 8, 54; *p* ≤ 0.05). Notably, cyantraniliprole had lower toxicity on *O. insidiosus* 7 DAT as compared with 3 DAT (Figure 9).

## 4. Discussion

In the past two decades, new classes of reduced-risk insecticides have been registered for insect pest control in many crops including cranberries; yet, few studies have compared the toxicity and residual activities of these insecticides on different life stages of pests. In this study, we found a high degree of specificity on the toxicities of reduced-risk insecticides on two key pests of cranberries, *S. sulfureana* and *Ch. parallela*, depending on insect stage (egg, larvae, and adult) and gender, and insecticide residual age. In general, only the spinosyn spinetoram and the organophosphate chlorpyrifos had broad-spectrum activities against all stages of the two lepidopteran pests tested here, while the remaining insecticides, that included organophosphate-replacement and reduced-risk chemistries, were more target specific in their toxicities. Moreover, as compared with the organophosphate chlorpyrifos, the newer insecticides had less negative effects on the predator *O. insidiosus*, indicating that these insecticides may be more compatible with biological control and can be implemented into IPM programs for the control of both pests.

Our experiments demonstrate high insecticidal and good residual (1–7 days) activities of the organophosphate chlorpyrifos and the spinosyn spinetoram against eggs, larvae, and adults of *S. sulfureana* and *Ch. parallela*. Insecticides affected egg hatch, but not their viability, indicating an effect at later stages of egg development. Both moth species protect their eggs with scales, which may reduce exposure of eggs to some insecticides; however, there were some species-specific differences in their susceptibility of eggs to insecticides (see below), indicating that other factors may also be involved. Charlet and Busaca [35] reported that chlorpyrifos applied at the proper time of the year prevents sunflower yield losses from the banded sunflower moth, *Cochylis hospes* Walsingham (Lepidoptera: Cochylidae). Chlorpyrifos is an insecticide intensively used in cranberries in New Jersey because of its broad-spectrum activity not just on chewing but also sucking insect pests; this insecticide is, however, under consideration for elimination by EPA in USA crops. Therefore, finding alternatives to chlorpyrifos is critical. The spinosyn spinosad was also toxic to all life stages (eggs, larvae, and adults) of the cranberry fruitworm *Acrobasis vaccinii* Riley (Lepidoptera: Pyralidae) [36]. Moreover, spinetoram had a lower LC_50_ value against the fall armyworm, *Spodoptera frugiperda* (J.E. Smith) (Lepidoptera: Noctuidae), larvae than the LC_50_s of seven other insecticides tested [37]. These studies together with ours demonstrate the broad-spectrum efficacy of spinosyns against different life stages of various lepidopteran pests; spinosyns can thus be used as alternatives to conventional broad-spectrum insecticides to manage *S. sulfureana* and *Ch. parallela*.

Two neonicotinoid insecticides were tested here: acetamiprid and thiamethoxam, but their efficacy depended on insect species and life stage. Acetamiprid had ovicidal activity on both *S. sulfureana* and *Ch. parallela*, while thiamethoxam was effective only on *Ch. parallela* eggs. Acetamiprid also had stronger adulticidal activity than thiamethoxam; thiamethoxam being toxic only to *S. sulfureana* males but not females, and moderately toxic to *Ch. parallela* adults. Both were weak against larvae, with acetamiprid tending to be more toxic than thiamethoxam and younger larvae being more susceptible to both insecticides than older instars. All together, these result indicate differential toxicities of insecticides that belong to the same class, with distinct contact toxicities on different insect species and stages. These differential toxic effects are likely due to the systemic nature of these insecticides [38], which might be influenced by their levels of penetration and persistence in insect and plant tissues [39]. For instance, Wise *et al.* [36] reported strong ovicidal and larvicidal, but weaker adulticidal, activities of the neonicotinoids acetamiprid and thiacloprid on *A. vaccinii* than broad-spectrum insecticides. They indicate that these neonicotinoids have relatively short-lived adult contact toxicity due to residue profiles on vegetable substrate; with declining toxicities as residues penetrate plant tissues. Yue *et al.* [40] also showed differential toxicities of neonicotinods depending on larval age, with higher concentrations and longer exposure periods of thiamethoxam required to provide 100% larval mortality as larvae of the Indianmeal moth, *Plodia interpunctella* (Hübner) (Lepidoptera: Pyralidae), age. Because of their systemic activity, neonicotinoids have mainly been used to target sucking insects [41]; our results show variable toxicities of this class of insecticides on two key lepidopteran pests of cranberries, which will likely limit their adoption.

The insect growth regulators (IGRs) methoxyfenozide (an ecdysone mimic [42]) and novaluron (a chitin inhibitor [43]) affected various *S. sulfureana* and *Ch. parallela* life stages differently. Methoxyfenozide was one of the most stage-specific insecticides tested, having weak ovicidal and adulticidal activity but excellent residual activity on 1st and 3rd instar larvae of both cranberry pests, indicating that this insecticide likely operated mainly via ingestion [42]. Stage-specific toxicity of methoxyfenozide was previously reported against *A. vaccinii* [36] and grape berry moth, *Paralobesia viteana* (Clemens) [44]. Wise *et al.* [36] also demonstrated that, although it does not have direct toxicity on eggs, methoxyfenozide showed ovi-larvicidal activity on *A. vaccinii* when applied after eggs were laid. Because methoxyfenozide is safe on bees, it is the only insecticide tested that can be used during bloom in cranberries, when *S. sulfureana* and *Ch. parallela* eggs are laid, so precise timing of spray applications is required for optimal control. The benzoylurea novaluron, on the other hand, had strong activity on *S. sulfureana* and *Ch. parallela* eggs, but weak adulticidal activity and moderate larvicidal, particularly on 1st instars, activity. Ovicidal activity of novaluron has been shown for lepidopteran (e.g., [36,45]), and coleopteran (e.g., [46,47]), pests. The use of the IGR novaluron as an ovicidal-larvicidal insecticide, as well as neonicotinoids, in cranberries may be limited by their potential toxicity to bees (e.g., [48,49,50]).

The newer insecticides, the anthranilic diamides chlorantraniliprole and cyantraniliprole affect muscle contraction [51]. These insecticides had strong activity on young *S. sulfureana* and *Ch. parallela* larvae, but were weak on 3rd instars in both laboratory and extended laboratory experiments, in particular on those of *S. sulfureana*. Stage-specific mortality effects could be due to metabolic differences in detoxification enzymes, as well as their feeding habits as older instars of both pest species produce silk webs [30,31], which may reduce their exposure to insecticides; however, these possibilities remain to be explored. Furthermore, the effects of insecticides on larval mortality and mass were assessed after seven days of exposure to treated diet or foliage; thus, it is possibly that our bioassay did not reflect fully the effects of these insecticides on larvae and stronger negative effects on 3rd instars could be seen after a longer exposure. Slow larval death might not be problematic to growers if exposed larvae cease feeding as shown by the reduced mass gained by *Ch. parallela* fed diamide-treated foliage; however, this was not the case for *S. sulfureana*, where diamide-exposed larvae gained as much mass as the controls. Both diamide insecticides also showed adulticidal activity and, in the case of chlorantraniliprole, ovicidal activity on *Ch. parallela*, suggesting exposure through contact in addition to via ingestion [52,53]. Most adults exposed to diamides were found alive but in a moribund state, indicating a slow-acting mode of action of this class of insecticides on both older instar larvae and adults, which is consistent with their negative effects on insect movement.

Interestingly, the oxadiazine insecticide indoxacarb had differential larval toxicity depending on pest species. It was toxic to *S. sulfureana* larvae but not to *Ch. parallela* larvae in both laboratory and extended laboratory experiments. This difference in toxicity between species is not likely due to resistance to indoxacarb in *Ch. parallela* because this insecticide has rarely been used to control this pest in cranberries in New Jersey; instead, physiological differences in levels of detoxification enzymes and/or behavioral differences in feeding habits are more possible explanations and worth exploring further. Indoxacarb had no ovicidal and only weak adulticidal activity. Liu *et al.* [54] also reported no toxic effects of indoxacarb on eggs of the diamondback moth, *Plutella xylostella* L., but a high efficacy, comparable to spinosad, against larvae. Unfortunately, the level of species-specificity of indoxacarb will likely limit its adoption for the control of these pests in cranberries, as has occurred thus far.

Reduced-risk insecticides are expected to be more compatible with biological control as compared with conventional broad-spectrum insecticides (e.g., [55]); yet, only a few studies have examined their non-target effects on natural enemies of agricultural pests to date [56]. In this study, we showed that organophosphate-replacement and reduced-risk insecticides were less toxic than the organophosphate chlorpyrifos on the insect predator *O. insidiosus*; however, all these newer insecticides, except for methoxyfenozide, had residual (three days) toxicities higher than the control. Similarly, Roubos *et al.* [57] showed high levels of toxicity of organophosphates on *O. insidiosus*; however, they also reported high mortality to acetamiprid and to exposure to fresh-residues of spinetoram, as well as moderate toxicities of indoxacarb and low toxicities of methoxyfenozide, novaluron, and chlorantraniliprole. The study by Roubos *et al.* [57] used insecticide-treated Petri dishes, whereas our bioassays were done with cranberry-treated foliage; *O. insidiosus* exposure to insecticides may thus vary depending on the type of treated substrate [32].

In summary, we have identified several insecticides that can be used as replacements to organophosphates (*i.e.*, chlorpyrifos) for *S. sulfureana* and *Ch. parallela* control in cranberries. Except for spinetoram, these newer insecticides had higher stage-specificity. Results from laboratory and extended laboratory experiments were consistent, indicating that substrate (diet *versus* foliage) did not influence larval exposure to the insecticides tested or their residual activity, which could be the case if insecticides are absorbed or broken down by plant tissues [58]. Both pest species overwinter as young larvae; thus, applications of insecticides such as chlorantraniliprole, methoxyfenozide, spinetoram, and cyantraniliprole, that are highly effective against this stage, should be applied early in the season if numbers exceed economic thresholds [17,30,31]. Fewer options are available later in the spring because older instars are more tolerant to some of these insecticides such as the diamides, so scouting and timing of insecticides are important. In the summer, 2nd generation larvae need to be controlled before they enter the fruit. Sex pheromones are available to monitor adult flight of both species [59,60,61], and for *S. sulfureana* a degree-day model is available to predict time of egg laying and hatch [62]. Among the insecticides tested here, methoxyfenozide is the only option during bloom in cranberries to prevent newly-hatched larvae from entering the fruit. After bloom, chlorantraniliprole, spinetoram, and cyantraniliprole could also be used as alternatives to chlorpyrifos (after cyantraniliprole is registered in cranberries). Although larvae are the main targets of insecticide applications due to their damage to fruit, spinetoram has the added benefit of having ovicidal activity, and chlorantraniliprole, spinetoram, and cyantraniliprole having adulticidal activity; these insecticides were also more compatible with biological control than organophosphates.

## 5. Conclusions

Our laboratory and extended laboratory experiments demonstrate that organophosphate-alternative and reduced-risk insecticides can be used for effective control of *S. sulfureana* and *Ch. parallela* in cranberries. These insecticides were, in general, more selective, *i.e.*, stage-specific, than conventional broad-spectrum insecticides, and will thus require more intensive scouting and precise timing of application. Implementation of these newer insecticides into current IPM programs will help reduce the reliance of growers on broad-spectrum insecticides (e.g., chlorpyrifos), while conserving biological control agents in cranberries. Moreover, these various classes of insecticides should be rotated pre- and post-bloom within and between seasons to prevent onset of resistant populations. In fact, organophosphate-resistant populations of *S. sulfureana* have been reported in cranberries in Massachusetts, USA [17].

## Figures and Tables

**Table 1:** List of insecticide names, classes, and rates used in laboratory and extended laboratory experiments.

Active Ingredient	Trade Name	Class	IRAC Group ^a^	Manufacturer	Rate (per Hectare) ^b^	Year(s) ^c^
Thiamethoxam ^d^	Actara 25WG	Nicotinoid	4A	Syngenta	0.28 kg	2015
Acetamiprid ^d,e^	Assail 30SG	Nicotinoid	4A	UPI	0.48 kg	2012
Spinetoram ^e^	Delegate 25WG	Spinosyn	5	Dow AgroSciences	0.42 kg	2012, 2013, 2014
Chlorantraniliprole ^e^	Altacor 35WDG	Diamide	28	DuPont	0.28 kg	2012, 2013, 2014
Cyantraniliprole ^e^	Exirel 10SE	Diamide	28	DuPont	0.95 kg	2012, 2013, 2014
Methoxyfenozide ^d,e^	Intrepid 2F	Insect Growth Regulator	18	Dow AgroSciences	1.17 L	2012, 2013, 2014
Novaluron ^d,e^	Rimon 0.8EC	Benzoylurea	15	Chemtura	0.88 L	2012
Indoxacarb ^d,e^	Avaunt 30WDG	Oxadiazine	22A	DuPont	0.42 kg	2012, 2015
Chlorpyrifos	Lorsban 4E	Organophosphate	1B	Dow AgroSciences	3.51 L	2012, 2013, 2014, 2015

^a^ Insecticide Resistance Action Committee; ^b^ Based on Oudemans *et al.* [29], except for Exirel 10SE that is not registered for cranberries (manufacturer recommendation); ^c^ Years when insecticide treatments were tested in extended laboratory experiments; ^d^ Organophosphate alternative [28]; ^e^ Reduced-risk insecticide [28].

**Table 2:** Results from analysis of variance (ANOVA) for laboratory studies on the effects of insecticides, days after treatment, instar (1st *versus* 3rd), and gender on *Sparganothis sulfureana* and *Choristoneura parallela* percent egg viability and hatch, larval mortality, and adult knockdown (moribund) and mortality.

Life Stage	Variables	*S. sulfureana*			*Ch. Parallela*		
Factor		*df*	*F*	*p* ^1^	*df*	*F*	*p* ^1^
**Eggs**							
Viability	Treatment	9, 90	1.70	0.09	9, 60	1.37	0.221
Hatch	Treatment	9, 90	15.79	**≤0.001**	9, 60	8.26	**≤0.001**
**Larvae**							
Mortality	Treatment	9, 360	33.71	**≤0.001**	9, 360	76.7	**≤0.001**
	Instar	1, 360	56.77	**≤0.001**	1, 360	19.4	**≤0.001**
	Date	1, 360	13.29	**≤0.001**	1, 360	4.29	**≤0.05**
	Treatment × Instar	9, 360	6.33	**≤0.001**	9, 360	5	**≤0.001**
	Treatment × Date	9, 360	5.18	**≤0.001**	9, 360	1.26	0.251
	Treatment × Instar × Date	9, 360	2.13	**≤0.05**	9, 360	0.54	0.842
**Adults**							
Moribund	Treatment	9, 240	11.45	**≤0.001**	9, 240	38.83	**≤0.001**
	Sex	1, 240	0.71	0.39	1, 240	0.12	0.723
	Date	2, 240	1.22	0.29	2, 240	1.9	0.15
	Treatment × Sex	9, 240	1.98	**≤0.05**	9, 240	0.4	0.932
	Treatment × Date	18, 240	3.60	**≤0.001**	18, 240	1.59	0.06
	Treatment × Sex × Date	18, 240	1.86	**≤0.05**	18, 240	1.87	**≤0.05**
Mortality	Treatment	9, 240	50.18	**≤0.001**	9, 240	40.46	**≤0.001**
	Sex	1, 240	11.20	**≤0.001**	1, 240	0.22	0.637
	Date	2, 240	11.20	**≤0.001**	2, 240	9.72	**≤0.001**
	Treatment × Sex	9, 240	3.58	**≤0.001**	9, 240	0.34	0.958
	Treatment × Date	18, 240	5.42	**≤0.001**	18, 240	2.19	**≤0.01**
	Treatment × Sex × Date	18, 240	0.41	0.98	18, 240	1.99	**≤0.05**

^1^ Numbers in bold indicate significant effects at α = 0.05.

**Table 3:** Results from analysis of variance (ANOVA) for extended laboratory studies on the effects of insecticides, days after treatment, instar, and their interactions on *Sparganothis sulfureana* and *Choristoneura parallela* percent larval mortality and mass of surviving 3rd instar larvae.

Species	Variables	*df*	*F*	*p* ^1^
Factor				
*S. sulfureana*				
Mortality	Treatment	9, 416	18.47	**≤0.001**
	Instar	1, 416	115.57	**≤0.001**
	Date	2, 416	35.09	**≤0.001**
	Treatment × Instar	9, 416	9.97	**≤0.001**
	Insecticide × Date	14, 416	1.74	**≤0.05**
	Insecticide × Instar × Date	14, 416	3.15	**≤0.001**
Mass	Treatment	9, 78	4.35	**≤0.001**
	Date	1, 78	0.60	0.44
	Treatment × Date	7, 78	1.36	0.23
*Ch. parallela*				
Mortality	Treatment	9, 459	36.94	**≤0.001**
	Instar	1, 459	166.90	**≤0.001**
	Date	2, 459	9.51	**≤0.001**
	Treatment × Instar	9, 459	6.12	**≤0.001**
	Insecticide × Date	15, 459	6.93	**≤0.001**
	Insecticide × Instar × Date	14, 459	2.64	**≤0.01**
Mass	Treatment	7, 59	10.01	**≤0.001**
	Date	1, 59	31.32	**≤0.001**
	Treatment × Date	4, 59	3.57	**≤0.05**

^1^ Numbers in bold indicate significant effects at α = 0.05.

## Abbreviations

The following abbreviations are used in this manuscript:

USA	United States of America
IPM	Integrated Pest Management
IRAC	Insecticide Resistance Action Committee
DAT	Days After Treatment
EPA	Environmental Protection Agency
ANOVA	Analysis of Variance
